# Limited Effects of Pain Control Treatments on Behaviour and Weight Gain of Pure and Crossbred Nellore Heifer Calves When Subjected to Hot-Iron Branding

**DOI:** 10.3390/ani12223143

**Published:** 2022-11-14

**Authors:** Adalinda Hernandez, Pedro Henrique Esteves Trindade, Mateus José Rodrigues Paranhos Da Costa, Jens Jung, Charlotte Berg

**Affiliations:** 1 Department of Animal Environment and Health, Swedish University of Agricultural Sciences, P.O. Box 234, SE–532 23 Skara, Sweden; 2 Departamento de Cirurgia Veterinária e Reprodução Animal, Faculdade de Medicina Veterinária e Zootecnia, UNESP, Universidade Estadual Paulista, Botucatu 14618-681, SP, Brazil; 3 Departamento de Zootecnia, Faculdade de Ciências Agrárias e Veterinárias, UNESP, Universidade Estadual Paulista, Jaboticabal 14884-900, SP, Brazil

**Keywords:** animal welfare, *Bos indicus*, cattle, facial expression, pain assessment, qualitative behavioural assessment, weight gain

## Abstract

**Simple Summary:**

This study investigated the effects of pain relief treatments on behaviour and weight gain in 92 Nellore and crossbred (F1 Aberdeen Angus × Nellore) heifer calves (aged around 120 days) subjected to hot-iron branding on the cheek after vaccination against brucellosis. Four treatments were compared: Control (CO, no pain relief); local anaesthesia (LA, subcutaneous anaesthetic); analgesic (LT, intramuscular meloxicam); and local anaesthetic and analgesic (LL, both local anaesthesia and meloxicam). Calves in treatments CO and LT were subjected to face manipulation simulating application of local anaesthetic without introducing the needle. Facial and body expressions, flight speed, and body weight were recorded before, during, and five (5-d) and 60 days (60-d) after hot-iron branding. No effect of pain relief was observed for most calves studied. The only difference observed was higher tension (‘tense’) at 5-d in CO calves. All calves showed fear and stress responses during restraint and hot-iron branding, which probably masked pain responses. In conclusion, hot-iron facial branding impoverishes calf welfare and, due to the additional handling involved, adoption of a pain relief protocol is not an efficient way to minimise welfare impacts.

**Abstract:**

Hot-iron branding is still commonly performed in cattle farming in tropical countries, and possibly has negative consequences for animal welfare and weight gain. This study examined the behavioural and weight gain responses of pure and crossbred Nellore heifer calves subjected to hot-iron branding on the cheek, without and with use of anaesthesia and analgesia. Ninety-two heifer calves, around 120 days old, were studied prospectively when subjected to hot-iron branding on the cheek (a statutory procedure in Brazil following brucellosis vaccination). Four randomly selected groups of calves were allocated to four treatments: no pain control (CO); subcutaneous anaesthetic local block (LA); intramuscular analgesia (meloxicam) (LT); and local anaesthesia plus meloxicam (LL). Behaviour, flight speed and body weight were evaluated before, during, and five (5-d) and 60 days (60-d) after branding. For these parameters, the only difference observed was higher tension in the CO group 5-d post-branding, suggesting a short-term negative effect of branding without pain control. The limited effects of the pain control treatments suggest interference in pain assessment by other factors, such as expression of fear and stress. Despite the lack of differences observed in behaviour and production parameters, facial hot-iron branding is an obvious welfare issue and, due to the additional handling involved, adoption of a simple pain relief protocol is not sufficient to minimise the welfare impact.

## 1. Introduction

Beef production is one of the main economic activities in Brazil [1] as well as in other parts of the world, and it is an important source of income for a large part of the population in tropical regions, including Latin America, Africa, and Asia [2]. However, certain routine management practices involve procedures that are painful and stressful for beef cattle, such as dehorning, castration, and hot-iron branding. These are usually carried out without any pain control treatment, such as anaesthetics, anti-inflammatory drugs, or analgesics [3,4,5,6,7]. These painful procedures usually result in traumatic experiences, generating stress and fear [8,9,10]. Therefore, there are negative consequences for animal welfare, which can lead to important changes in animal behaviour, compromising the human-animal relationship (HAR) [11]. Unpleasant emotions, such as stress, fear, and pain have been associated with negative impacts on HAR [12].

Fear of humans usually results in stress, and has a potential negative impact on production outcomes and cattle health [13]. When calves learn to associate humans with pain and discomfort, they are also likely to be more afraid of humans later, and hence more difficult to manage [14]. In contrast, gentle management tends to reduce aggressive behaviour in calves, even months after the management procedure [15]. For example, Becker and Lobato [14] and Probst et al. [13] observed shorter avoidance distances and fewer attempts to escape in calves that had received gentle handling in comparison with those that had not.

In Brazil, cattle farmers are required by the Ministry of Agriculture, Livestock and Supply (MAPA) to perform hot-iron branding on the cheek of heifer calves at the time of vaccination against brucellosis [16], and this procedure is invariably performed without using any kind of pain control. Studies in rodents have shown that painful procedures in young animals predispose them to higher pain sensitivity, anxiety, increased fear expressions and passive coping behaviours [9]. There is a lack of similar studies in cattle, but previous studies have shown that hot-iron branding can result in severe pain [17,18,19,20], and thus possibly has long-term psychological consequences.

Pain-related behavioural expression has been extensively investigated in cattle using species-specific indicators, e.g., body posture, movements, and facial expression [21,22,23,24]. Negative consequences of pain on production parameters (e.g., weight gain) have also being investigated, with the results showing that when pain relief is used during painful procedures, such as castration or dehorning, beef calves have greater weight gain [4,25,26].

The aim of this study was to investigate the behavioural and weight gain responses of pure and crossbred Nellore heifer calves experiencing hot-iron branding on the cheek, with and without use of pain relief comprising local anaesthesia, an anti-inflammatory drug, or a combination of both.

## 2. Materials and Methods

Data collection was performed on a commercial farm located in the municipality of Araguaiana, Mato Grosso State, Brazil. This farm has around 1300 Nellore cows, producing pure and crossbred (F1 Aberdeen Angus × Nellore) calves. In compliance with MAPA Normative Instruction Nº 19 of 10 October 2016 [15], all heifer calves on the farm are vaccinated against brucellosis at around 120 days of age. At the same time, a hot-iron brand, showing the final digit of the year of vaccination, is applied to the cheek of each calf. In this study, 92 heifer calves were evaluated, 32 pure Nellore and 60 crossbreeds. To minimise the disturbance to farm routines, the baseline assessments and immediate post-branding assessments were performed on three groups (32 Nellore, 30 crossbreeds, 30 crossbreeds), each on a different day. No calf was branded exclusively for the purposes of this study.

At the beginning of the study, all selected heifer calves were individually assessed for body condition and health. They were then divided by stratified randomisation into four groups, with 23 heifer calves per group, and allocated to one of the following experimental treatments: control (CO), branded in the conventional way, without any procedure to relieve pain; local anaesthetic (LA), with a subcutaneous 5-mL local anaesthetic block consisting of 75% lidocaine (2% Xylestesin^®^, Cristalia) and 25% bupivacaine (0.5%, Neocaína^®^, Cristalia), both without vasoconstrictor, injected in the centre of the area where the hot-iron brand would be placed; long-term analgesia (LT), with a intramuscular dose of 0.5 mg/kg body weight of the long-term anti-inflammatory and analgesic drug meloxicam (2%, Maxicam^®^, Ourofino) before branding; and local anaesthetic plus long-term analgesia (LL), combining the LA and LT treatments. Heifer calves in treatments CO and LT were subjected to face manipulation simulating application of local anaesthetic, but without introducing the needle.

Restraint and hot-iron branding were carried out by experienced livestock staff and a veterinarian was responsible for drug administration. The animals were branded 10–15 min after drug administration/simulation. All procedures were carried out with the heifer calves inside a squeeze chute, using a head bail.

The body weight of all calves was recorded with an electronic cattle scale (True-test KitS3 + mp600). An electronic device (DUBOI^®^, Campo Grande, MS, Brazil), composed of two pairs of photoelectric cells, a stopwatch, and a small processor, was installed in the corridor at the exit of the squeeze chute to record the time taken by each calf to cover a distance of 2 m. The value obtained was used to calculate the flight speed (FS) of each calf, as described by Burrow et al. [27].

Body weight and FS were recorded three times, at baseline (one hour before starting handling procedures for branding), and 5 and 60 days after hot-iron branding. An extra (fourth) measurement of FS was taken just after branding. Two cameras were used to record calf behaviour, one filming the head of the calf at a 90° angle and other the body from above and behind, at a 45° angle. The animals were recorded during one minute on four different occasions: At baseline, at branding (during and immediately after branding), and at 5 and 60 days after hot-iron branding. The video recordings were studied to assess calf behaviour in terms of body reactions and facial expressions (as described in Table 1 and Table 2, respectively). Additionally, qualitative behaviour assessment (QBA), adapted from Wemelsfelder et al. [28], was performed to assess the emotional state of the calves, using a visual analogue scale with seven terms: calm, fearful, agitated, tense, comfortable, painful, and stressed. Behavioural and QBA assessments were carried out by two trained observers with previous experience of these, with QBA and facial expression behaviours always assessed by one of these observers and body reactions always assessed by the other. Intra-observer reliability tests were carried out twice, with an interval of 30 days. In these, the observers assessed 20 videos from all experimental groups, without knowing the treatment group to which the calves belonged.

### Statistical Analyses

All statistical analyses were performed using the software RStudio (version 1.0.143), with an alpha value of 5% considered significant. Intra-observer reliability was assessed using weighted kappa coefficient (function “cohen.kappa” in the R “psych” package) for scores and frequencies. An intra-class correlation coefficient of the kind “agreement” was applied to qualitative behaviour assessment (QBA) terms. To account for missing data, a mean (normal distributed variables) or median (non-normal distributed variables) estimate was made for the experimental group and sampling occasion.

Statistical analyses were carried out in two steps. First, behavioural and body weight changes over time (baseline, immediately after branding, and 5 and 60 days after branding) within treatments (CO, LA, LT and LL) were assessed. Normality was tested with the Shapiro-Wilk test (function “shapiro.test” in the R “stats” package). Flight speed and body weight showed a normal distribution, and mixed linear models for repeated measures (function “lmer” of the R “lme4”; package) were used. For dichotomous variables (eye white showing, third eyelid, grunting), logistic regression analysis (“glm” function in the “stats” package) was applied. The other variables were assumed to have a non-normal distribution, and mixed generalised models for repeated measures (function “glmer” in the R “lme4” package) were used. All models considered sampling occasion and genetic group as fixed effects, and calf as random effect. The Bonferroni procedure was used as a post-hoc test to correct for probability of rejection of the null hypothesis (function “lsmeans” in the R “lsmeans” package).

Second, comparisons between treatments were carried out for each sampling occasion. For FS and body weight, analysis of variance (function “aov” of the R “stats” package) was performed, considering treatment and genetic group as fixed effects, with the Bonferroni procedure used as a post-hoc test. For the dichotomous variables, logistic regression analysis was performed (“glm” function in the “stats” package) using the model described above. Finally, for the non-normal variables, a Kruskal–Wallis test (function “kruskal” of the R “agricolae” package) was carried out to compare the effects of treatments.

## 3. Results

The intra-observer reliability was high for all behavioural (body reactions and facial expressions) and QBA variables, with weighted kappa coefficient ranging in value from 0.72 to 1.00 (all statistically significant) (Table 3).

**Figure 1:**
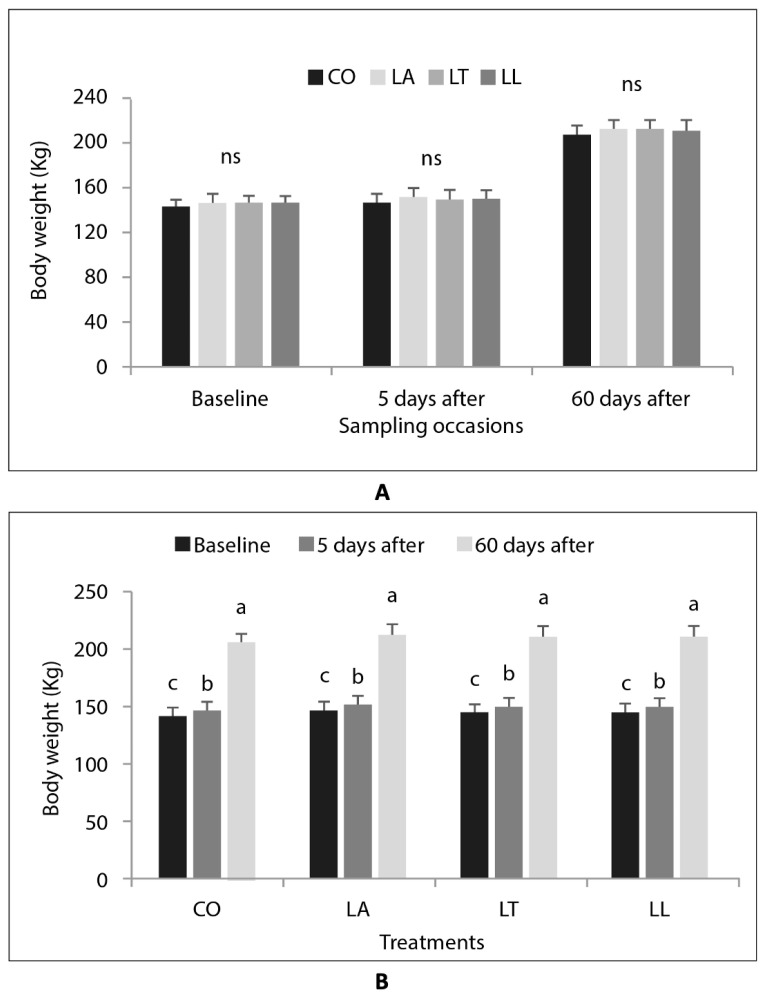
Body weight (kg) of heifer calves in the four treatments (CO = control group; LA = local anaesthetic; LT = intramuscular analgesic; LL = local anaesthetic + intramuscular analgesic) at different sampling occasions (baseline, 5 days, and 60 days after branding), compared by (**A**) occasion and (**B**) treatment. Mean values, lines on bars show standard error. Different letters (a > b) indicate statistically significant difference between treatments within sampling occasion, ns = no significant difference.

No significant difference between treatments was found for body weight (Figure 1A). However, as expected due to calf growth, the sampling occasion had an expressive effect on body weight, with an increase over time in all treatments, resulting in the highest mean values at 60 days after branding (Figure 1B).

**Figure 2:**
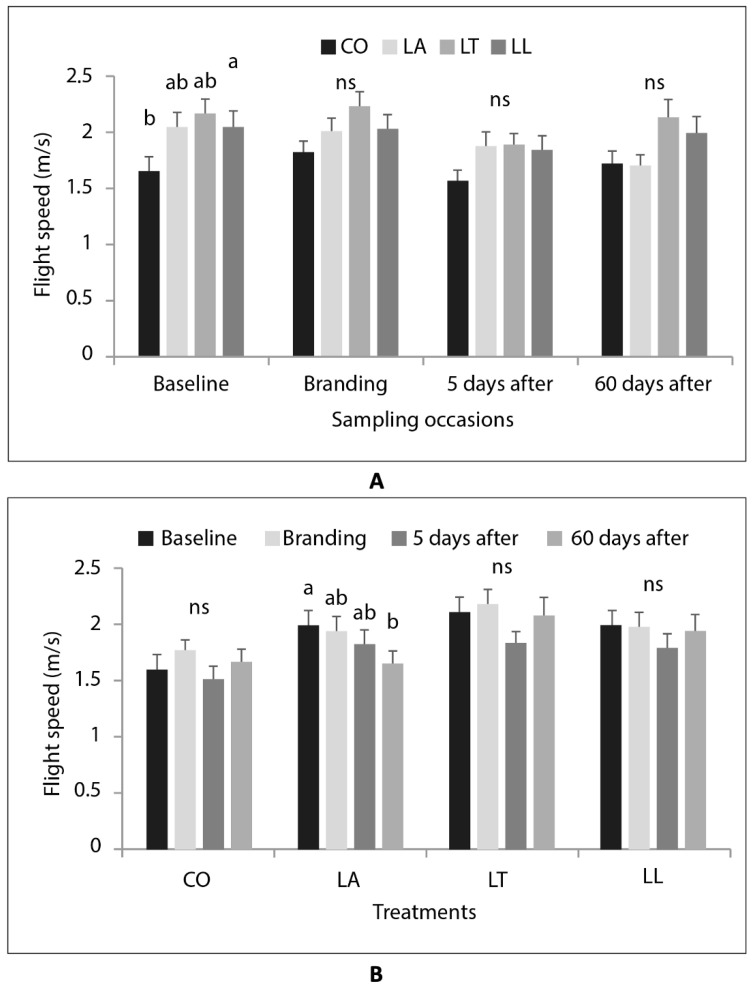
Flight speed (m/s) of heifer calves in the four treatments tested (CO = control group; LA = local anaesthetic; LT = intramuscular analgesic; LL = local anaesthetic + intramuscular analgesic) at different sampling occasions (baseline, branding, and 5 days and 60 days after branding), compared by (**A**) occasion and (**B**) treatment). Mean values, lines on bars show standard error. Different letters (a > b) indicate statistically significant difference between treatments within sampling occasion, ns = no significant difference.

Flight speed differed significantly between the treatments only during baseline data collection, with LT (highest mean FS) differing significantly from CO, but not from LA and LL (Figure 2A). Sampling occasion had a significant effect on FS only in calves in the LA treatment, which showed a progressive reduction in FS over time (Figure 2B), with the highest mean value being observed at baseline and the lowest at 60 days after branding (*p* < 0.05).

Of all the body reaction variables studied, only movement scores showed significant differences between procedures, and these occurred only during baseline assessment, with LL showing a higher mean value than LA, but both did not differ from the CO and LT means (Table 4). In contrast, tail position/movement score was different over time, CO scored higher at the time of branding compared to 5 and 60 days after branding; LA scored higher during branding than on all the other sampling occasions; LL scored higher at the time of branding in relation to 5 days after branding; and LT scored higher at branding compared to baseline. (Table 4). The percentage distribution of scores for all body reaction variables are shown in Supplementary Table S1.

Among the facial expression variables studied, only ‘tension of masticatory muscle’ differed significantly between treatments at 5 days after branding, with CO showing the highest mean value but not differing from LL and LT. In addition, sampling occasion had a significant effect on ‘eye tightness’ and ‘opening mouth’ within CO, with higher mean values for ‘eye tightness’ being observed at branding and 5 days after, and the highest mean for ‘opening mouth’ observed during branding, although it did not differ significantly from the mean value obtained at 60 days after branding (Table 5). ‘Frequency of swallowing’ also showed significant differences between sampling occasions, with highest mean value observed at branding (Table 5). The percentages of heifer calves that swallowed or vocalised (screamed/grunted) in the different treatments and the different sampling occasions are shown in Table 6. The percentage distribution of scores for all facial expression variables are shown in Supplementary Table S2.

There was a significant effect of treatment on QBA ‘tense’ ratings only for the measurements made at 5 days after branding, with LA showing the lowest value of all treatments (Table 7). The mean rating for ‘fearful’ was higher at the baseline assessment compared with all other sampling occasions within the CO, LA, and LL treatments. A similar result was found for ‘comfortable’, but restricted to the CO treatment, with the highest mean value also found at baseline, which did not differ from 60 days after branding. Sampling occasion had a significant effect on ‘painful’ within all treatments, with the highest mean values always being found at branding. Finally, the mean value of ‘agitated’ differed significantly between sampling occasions within the LT treatment, with the lowest value being found at branding (Table 7).

## 4. Discussion

Painful procedures are still a major welfare issue on beef cattle farms. When performed without any pain control, these procedures often lead to traumatic experiences for the animals [8,9,10,30], generating fear and leading to behavioural changes that can possibly impair the human-animal relationship [11,31,32,33].

Apart from some differences between pure and crossbred Nellore heifer calves and lower body weight in the control group (CO) from the baseline onwards due to randomisation, there were no significant effects of the different treatments on body weight in the present study. This could indicate that branding with pain relief does not make a substantial difference during the procedure compared with branding without pain relief. In contrast, Tucker et al. [34] found that use of a nonsteroidal anti-inflammatory drug (flunixin) can improve weight gain in cattle in the days after hot-iron branding. However, Schwartzkopf-Genswein et al. [35] concluded that branding alone may not be a sufficiently severe event to have a negative effect on weight gain in cattle.

Likewise, the results in the present study did not show clear behavioural differences between the treatments at the moment of branding. This could be partly explained by the young age of the heifer calves studied (120 days) and their lack of previous experiences in a squeeze chute, resulting in stress responses. Although the level of pain may have been different between treatments, the level of fear and stress during this new unpleasant experience could have been similar for all animals, regardless of treatment. In addition, expression of acute pain and expression of stress or fear caused by the whole handling and restraining situation could have been similar. In previous studies measuring pain responses in cattle, handling and restraint have been suggested to interfere with pain expression [17,18]. Such interference apparently masked possible differences between treatments in the present study. This assumption is consistent with the lack of differences observed in flight speed measurements between treatments. In contrast, Müller et al. [36] observed that flight speed can increase due to a rise in fearfulness. However, individual differences in the way in which calves react to stressful situations should be considered, since according to Grandin and Shivley [37], “the animal’s response is highly dependent on both its previous experience and inherited traits, such as temperament”. The heifer calves assessed in the present study had no previous experience of handling in the squeeze chute, and differences in temperament may have resulted in some heifer calves reacting more than others to this new experience, e.g., by running away and jumping as opposed to freeze reactions. These two responses have been observed to occur within *Bos indicus* cattle subjected to stressful situations [35], which may explain the lack of significant differences in flight speed between treatments. Another factor contributing to the lack of significant differences in flight speed between treatments in the present study could be that a difference in fight speed was observed from the baseline, and this may have masked to the differences during and after branding.

Nevertheless, the results of this study indicate that the heifer calves which did not receive any kind of pain relief (CO) may have been more tense when they returned to the squeeze chute five days after the procedure, suggesting that the use of pain control gave a short-term positive effect. However, this effect was probably not long-lasting, as at 60 days after branding no medium-term effect on behaviour was observed and reactions of fear and stress were similar in all experimental groups. Müller et al. [38] reported facial expressions of pain during and after facial hot-iron branding, but the use of effective methods to control the pain caused by more than one hot-iron branding on the back quarter of adult bovines has not been studied thoroughly. However, it has been reported that a single injection of a non-steroidal anti-inflammatory drug does not result in measurable pain relief [35], which is consistent with findings in the present study.

Assessment of the animals in this study was essentially behavioural, and greater differences between treatments might be observed on assessing changes in other traits, such as pain and stress biomarkers. In a recent study, Martin et al. [39] found that use of meloxicam resulted in differences in infrared thermography and reduced lying bouts in Hereford and Angus calves after branding on the hip with an electric iron.

There are few comparative studies measuring cortisol levels in cattle during hot-iron branding with and without pain relief. Studies addressing other painful procedures, such as dehorning and disbudding, have found differences in plasma cortisol responses when a good pain relief protocol is applied e.g., [31,40]. It could be more difficult to achieve similar results when applying anaesthetic protocols for facial or body hot-iron branding, which involved infiltration anaesthesia in this study, but for dehorning and disbudding often involves blocking [41,42]. However, Bates et al. [43], found that “the local infiltration block method appears to represent a viable alternative to the cornual (nerve) block method” when disbudding calves.

Injecting anaesthetics and analgesics may not be perceived as practicable in commercial farming, as it means extra costs for the drugs, equipment, and administration time required [44]. The need to hire a veterinarian to administer the injections correctly could also pose a problem for remote farms and farms with limited access to veterinary services. Injecting drugs into the animals could also interfere with the private animal health and welfare standards of beef production on some markets e.g., [44]. The resistance to adopting anaesthetic protocols is likely to be even greater in large herds, where up to 300 heifer calves are commonly vaccinated against brucellosis and hot-iron branded in a working day (personal information). Additionally, use of anaesthesia prior to branding would require double handling within a short period, with the inevitable consequence of increasing the handling time and stress for the animals, in itself constituting a welfare issue [45].

In general, performing compulsory branding of heifer calves after vaccination for brucellosis poses a high risk to the handlers, since one person must hold the calf’s head in their hands (often bare), keeping them very close to the site where the hot-iron is applied. Moreover, during the entire session the handlers will breathe in smoke from the brazier where the iron is heated and from burning animal hair and skin. The stipulated branding site is also situated close to the animal’s eye, adding an increased discomfort and risk of accidents for the animals.

In light of the above, we argue that facial hot-iron branding should be completely phased out, rather than handled in terms of pain relief, as the practice compromises animal welfare and is not a necessary or efficient way of ensuring proper vaccination status. Alternative identification (ID) methods should be applied [46] and further research and innovation is needed to find affordable and less invasive alternatives. In the USA, where hot-iron branding on the cheek was formerly required after vaccinating against brucellosis, the procedure has now been replaced by an ear tattoo, which is less painful and easier to apply [47].

It is important to note that the farm where the present study was performed had adequate facilities to manage the animals and provided training for the workers, who were experienced and did not show any negative interaction with the animals. This is not necessarily the case on all farms, and if there are substantial differences in conditions, different results might be obtained. Squeeze chute design may also differ between farms and there may be better chutes available, as that used in this study was designed for adult cattle and not for smaller animals such as heifer calves. In any case, more research is needed on traumatic effects of handling on the animal (related to mental wellbeing and human-animal interactions) and on the effects of iron branding on different parts of the body and under different restraining conditions.

## 5. Conclusions

Based on the findings in this study, it can be concluded that hot-iron facial branding represents an obvious welfare issue, not only through the painful procedure itself, but also through the whole restraining and fearful situation, with the animals showing an obvious negative reaction. There are also concerns about labour safety and discomfort that should be considered. Our results indicate that these problems cannot easily be solved by applying a simple pain control protocol. Hence, facial hot-iron branding should be completely phased out, rather than handled in terms of pain relief, as the practice compromises animal welfare. It is also not an efficient way of confirming vaccination status and alternative identification methods should be developed. Further research and innovation are needed to find affordable and less invasive alternatives. Further studies under slightly different management situations should also be performed, to investigate whether the discomfort observed here was related to pain, fear, or the restraint itself. Other metrics, such as stress hormones or heart rate, should be considered for more accurate assessment of the differences between treatments.

## Figures and Tables

**Table 1:** Definitions of the scores for body reactions used as indicators of pain and stress in heifer calves subjected to hot-iron branding on the cheek after brucellosis vaccination.

Body Reaction	Score	Definition
Movement (frequency and intensity of movement of the calf while restrained in the squeeze chute, adapted from Grandin [29])	0	No movement.
	1	Little movement, during less than half the observation time.
	2	Frequent movements (during half the observation time or more), but not vigorous.
	3	Constant and vigorous movements.
	4	Constant and vigorous movements: animal jumps and raises the limbs off the ground.
Tail position/movement	0	Relaxed: tail of the animal in a neutral position, pointing down and showing natural movements during the whole observation time.
	1	Stiff, pointed down: tail pointing down at 45° and completely stiff (behaviour clearly displayed at least once).
	2	Flicking: tail showing intense movements involving the whole extremity from base to tip (behaviour clearly displayed at least once).
	3	Between the hind legs: tail held completely stiff between the legs of the animal (behaviour clearly displayed at least once).
	4	Shaking: base of the tail motionless, with the tip showing frequent, but not intense, movements (behaviour clearly displayed at least once).
Hind leg movement	0	All four feet are on the ground and the calf does not show intense movements with the legs during the whole observation time.
	1	One hind leg is on the ground and the other is lifted repeatedly (stepping) (behaviour clearly displayed at least once).
	2	One of the hind legs is intensely moving sideward or backward (kicking) (behaviour clearly displayed at least once).
	3	Both hind legs are intensely moving sideward or backward, raising the limbs off the ground (jumping) (behaviour clearly displayed at least once).
Movement of back/spine	0	No movement: the spine is in a relaxed position in a 90° angle during the whole observation time.
	1	Little movement: the spine is not completely relaxed, but not arching entirely (behaviour clearly displayed at least once).
	2	Arched spine: the spine of the animal is bent in an arched position (behaviour clearly displayed at least once).
Reaction at release	0	Walking.
	1	Standing still for a moment before walking out.
	2	Running.
	3	Jumping out.
Body response to branding *	0	No response.
	1	Subtle response (little movement).
	2	Clear response (jumping, kicking, or screaming).

* Assessed only at the instant of hot-iron branding.

**Table 2:** Definitions of the scores for facial expressions used as pain and stress indicators in heifer calves subjected to hot-iron branding on the cheek after brucellosis vaccination. Rating scale adapted from Gleerup et al. [22].

Facial Expression	Score	Definition
Head position	0	Relaxed, no movement during video recording.
	1	Pushing forward or backward against the wood at least once during video recording.
	2	Pushing upward at least once during video recording.
Escape reaction	0	Not trying to escape from the squeeze chute during video recording.
	1	Trying to escape from the squeeze chute with moderate movements during most video recording.
	2	Trying to escape from the squeeze chute with intense movements during most video recording.
Eye white showing	0	Sclera not visible during video recording.
	1	Sclera visible at least once during video recording.
Tension above eye	0	No tension above eye during video recording.
	1	Moderate tension above eye during most video recording.
	2	High tension above eye during most video recording.
Eye tightness	0	Closing the eyes without twitching eyelids when blinking.
	1	Closing the eyes when blinking by moderately contracting the eyelids at least once during video recording.
	2	Closing the eyes when blinking by intensely contracting the eyelids at least once during video recording.
Third eyelid	0	Third eyelid not visible during video recording.
	1	Third eyelid visible at least once during video recording.
Tension in the masticatory muscles	0	No tension in the masticatory muscle during video recording.
	1	Moderate tension in the masticatory muscle during most of video recording.
	2	High tension in the masticatory muscle during most of video recording.
Tension of the muzzle	0	No muzzle tension during video recording.
	1	Moderate muzzle tension during most of video recording.
	2	High muzzle tension during most of video recording.
Opening mouth	0	Mouth closed during video recording.
	1	Mouth open at least once during video recording.
	2	Mouth open with tongue sticking out at least once during video recording.
Swallowing	--	Frequency of something passing down the throat.
Vocalisation	--	Frequency of screaming/grunting
Face response to branding *	0	No reaction to the hot-iron during video recording.
	1	Moving head against the hot-iron at least once during video recording.

* Assessed only during the time of hot-iron brand placement.

**Table 3:** Intra-observer reliability (weighted kappa and interclass agreement coefficients) and respective confidence intervals of body reactions, facial expressions, and qualitative behaviour assessment variables.

	Weighted Kappa	95% Confidence Interval	
Variable	Coefficient	Minimum	Maximum
Body reaction			
Movement	0.92	0.86	0.99
Tail position/movement	0.96	0.9	1.00
Hind legs movement	0.91	0.83	1.00
Movement of back/spine	1.00	1.00	1.00
Reaction at release	0.79	0.52	1.00
Reaction to branding			
Facial expression			
Head position	0.88	0.73	1.00
Escape reaction	0.72	0.38	1.00
Eye white showing	1.00	1.00	1.00
Tension above eye	0.78	0.49	1.00
Eye tightness	0.89	0.72	1.00
Third eyelid	1.00	1.00	1.00
Tension of the masticatory muscles	0.88	0.75	1.00
Tension of the muzzle	0.88	0.72	1.00
Opening mouth	1.00	1.00	1.00
Swallowing	0.94	0.83	1.00
Vocalisation (screaming/grunting)	1.00	1.00	1.00
Face response to branding	1.00	1.00	1.00
	**Intraclass agreement**	**95% confidence interval**	
**Variable**	**coefficient**	**Minimum**	**Maximum**
Qualitative behaviour assessment			
Calm	0.84	0.65	0.93
Fearful	0.77	0.50	0.90
Agitated	0.79	0.55	0.91
Tense	0.88	0.74	0.95
Comfortable	0.73	0.44	0.88
Painful	0.95	0.89	0.98
Stressed	1.00	1.00	1.00

**Table 4:** Body reaction score for heifer calves in the four procedures (CO = control; LA = local anaesthetic; LT = long-term intramuscular analgesic; LL = local anaesthetic + intramuscular analgesic) on different sampling occasions (baseline, branding, and 5 days and 60 days after branding). Mean ± standard error (SE), only variables that differed significantly (*p* < 0.05) between treatments, or over time within treatments, are shown. Lowercase letters indicate significant difference over time within treatments (a > b > c > d), capital letters indicate significant difference between treatments (A > B > C > D).

Body Reaction	Sampling Occasion			
	Baseline	Branding	5 Days after	60 Days after
	(Mean ± SE)	(Mean ± SE)	(Mean ± SE)	(Mean ± SE)
CO				
Movement	0.83 ± 0.19 ^AB^	0.79 ± 0.21	0.66 ± 0.19	0.73 ± 0.20
Tail position/movement	1.73 ± 0.19 ^ab^	2.79 ± 0.18 ^a^	1.17 ± 0.17 ^b^	1.61 ± 0.15 ^b^
LA				
Movement	0.48 ± 0.17 ^B^	0.52 ± 0.14	0.48 ± 0.14	0.79 ± 0.18
Tail position/movement	1.35 ±0.14 ^b^	2.83 ± 0.20 ^a^	1.47 ± 0.17 ^b^	1.43 ± 0.12 ^b^
LL				
Movement	1.27 ± 0.24 ^A^	0.96 ± 0.21	0.61 ± 0.17	0.96 ± 0.28
Tail position/movement	1.83 ± 0.15 ^ab^	2.61 ± 0.19 ^a^	1.35 ± 0.11 ^b^	1.53 ± 0.13 ^ab^
LT				
Movement	0.91 ± 0.21 ^AB^	0.86 ± 0.23	0.73 ± 0.20	0.83 ± 0.19
Tail position/movement	1.47 ± 0.15 ^b^	2.65 ± 0.22 ^a^	1.61 ± 0.13 ^ab^	1.65 ± 0.17 ^ab^

**Table 5:** Facial expression scores (S) and frequencies (F) for heifer calves in the four treatments (CO = Control group; LA = local anaesthetic; LT = long-term intramuscular analgesic; LL = local anaesthetic + intramuscular analgesic) on different sampling occasions (baseline, branding, and 5 days and 60 days after branding). Mean ± standard error (SE), only variables that differed significantly (*p* < 0.05) between treatments, or over time within treatments, are shown. Lowercase letters indicate significant difference over time within treatments (a > b), capital letters indicate significant difference between treatments (A > B).

Facial Expression	Sampling Occasion			
	Baseline	Branding *	5 Days after	60 Days after
	Mean ± SE	Mean ± SE	Mean ± SE	Mean ± SE
CO				
Eye tightness (S)	0.96 ± 0.20 ^a^	1.13 ± 0.16 ^a^	0.96 ± 0.17 ^a^	0.27 ± 0.12 ^b^
Tension masticatory muscles (S)	1.09 ± 0.15	1.21 ± 0.18	1.57 ± 0.12 ^A^	0.91 ± 0.17
Opening mouth (S)	0.08 ± 0.06 ^b^	0.77 ± 0.17 ^a^	0.17 ± 0.11 ^b^	0.21 ± 0.10 ^ab^
LA				
Tension masticatory muscles (S)	1.22 ± 0.14	0.66 ± 0.17	0.69 ± 0.13 ^B^	0.86 ± 0.15
LL				
Tension masticatory muscles (S)	1.09 ± 0.17	1.00 ± 0.18	0.96 ± 0.20 ^AB^	1.05 ± 0.17
Swallowing (F)	0.66 ± 0.13 ^b^	1.57 ± 0.71 ^a^	0.69 ± 0.14 ^b^	0.61 ± 0.10 ^b^
LT				
Tension masticatory muscles (S)	1.17 ± 0.13	0.79 ± 0.14	1.09 ± 0.18 ^AB^	0.79 ± 0.15

* Assessed immediately after branding.

**Table 6:** Percentage of heifer calves in the four procedures (CO = Control group; LA = local anaesthetic; LT = long-term intramuscular analgesic; LL = local anaesthetic plus intramuscular analgesic) that swallowed or vocalised (screamed/grunted) on different sampling occasions (baseline, branding, and 5 days and 60 days after branding).

Treatment				
Facial Expression	Baseline (%)	Branding (%)	5 Days after (%)	60 Days after (%)
CO				
Swallowing	43.5	52.2	34.8	60.9
Vocalisation (screaming/grunting)	0.0	26.1	4.4	0.0
LA				
Swallowing	54.6	54.6	56.5	30.4
Vocalisation (screaming/grunting)	4.6	22.7	0.0	0.0
LL				
Swallowing	59.1	69.6	56.5	60.9
Vocalisation (screaming/grunting)	4.6	26.1	0.0	0.0
LT				
Swallowing	36.4	69.6	52.2	47.6
Vocalisation (screaming/grunting)	0.0	17.4	0.0	4.8

**Table 7:** Qualitative behaviour assessment (QBA) ratings (visual analogue scale, cm) for heifer calves in the four treatments (CO = control group; LA = local anaesthetic; LT = long-term intramuscular analgesic; LL = local anaesthetic + intramuscular analgesic) on different sampling occasions (baseline, branding, and 5 days and 60 days after branding). Mean ± standard error (SE), only variables that differed significantly (*p* < 0.05) between treatments, or over time within treatments, are shown. Lowercase letters indicate significant difference over time within treatments (a > b), capital letters indicate significant difference between treatments (A > B).

QBA Term	Sampling Occasion			
	Baseline	Branding	5 Days after	60 Days after
	Mean ± SE	Mean ± SE	Mean ± SE	Mean ± SE
CO				
Fearful	4.23 ± 0.40 ^a^	2.59 ± 0.45 ^b^	2.49 ± 0.41 ^b^	2.13 ± 0.28 ^b^
Tense	5.93 ± 0.37	6.43 ± 0.41	7.06 ± 0.32 ^A^	6.20 ± 0.37
Comfortable	1.07 ± 0.24 ^a^	0.28 ± 0.09 ^b^	0.34 ± 0.07 ^b^	0.66 ± 0.17 ^ab^
Painful	0.69 ± 0.18 ^b^	4.81 ± 0.39 ^a^	0.67 ± 0.15 ^b^	0.29 ± 0.09 ^b^
LA				
Fearful	3.50 ± 0.50 ^a^	1.63 ± 0.27 ^b^	2.66 ± 0.38 ^ab^	2.17 ± 0.35 ^b^
Agitated	3.35 ± 0.59 ^ab^	2.15 ± 0.42 ^b^	4.23 ± 0.57 ^a^	3.59 ± 0.55 ^a^
Tense	5.92 ± 0.29	5.30 ± 0.36	5.07 ± 0.30 ^B^	6.23 ± 0.30
Painful	0.73 ± 0.20 ^b^	3.52 ± 0.31 ^a^	0.31 ± 0.08 ^b^	0.21 ± 0.08 ^b^
LL				
Fearful	3.85 ± 0.54 ^a^	1.81 ± 0.27 ^b^	2.67 ± 0.49 ^ab^	2.01 ± 0.23 ^b^
Tense	6.21 ± 0.39	5.81 ± 0.42	6.53 ± 0.31 ^A^	6.06 ± 0.44
Painful	0.59 ± 0.12 ^b^	4.53 ± 0.45 ^a^	0.36 ± 0.08 ^b^	0.31 ± 0.07 ^b^
LT				
Agitated	3.77 ± 0.44 ^ab^	3.00 ± 0.45 ^b^	4.83 ± 0.51 ^a^	3.93 ± 0.57 ^ab^
Tense	5.61 ± 0.36	5.81 ± 0.47	6.45 ± 0.38 ^A^	6.32 ± 0.32
Painful	0.54 ± 0.13 ^b^	4.07 ± 0.34 ^a^	0.57 ± 0.16 ^b^	0.21 ± 0.06 ^b^

## Data Availability

The authors confirm that the data supporting the findings of this study are available within the article and its supplementary materials.

## Supplementary Materials

The following supporting information can be downloaded at: https://www.mdpi.com/article/10.3390/ani12223143/s1, Table S1: Percentage distribution of body reaction category scores 0–4 (see Table 1) in the four treatments (CO = control group; LA = local anaesthetic; LT = intramuscular analgesic; LL = local anaesthetic plus intramuscular analgesic) on different sampling occasions (baseline, branding, and 5 days and 60 days after branding); Table S1: Percentage distribution of facial expression category scores 0–2 (see Table 2) in the four treatments (CO = Control group; LA = local anaesthetic; LT = intramuscular analgesic; LL = local anaesthetic plus intramuscular analgesic) on different sampling occasions (baseline, branding, and 5 days and 60 days after branding).
