# Supplementary material for: Limited Effects of Pain Control Treatments on Behaviour and Weight Gain of Pure and Crossbred Nellore Heifer Calves When Subjected to Hot-Iron Branding
Source: Animals (Basel). 2022 Nov 14;12(22):3143. doi: 10.3390/ani12223143 (PMC9686943; doi:10.3390/ani12223143)
Supplement: Supplementary file 1 [file animals-12-03143-s001.zip › animals-1937109-supplementary.pdf]

Supplementary Files

Supplementary Table S1. Percentage distribution of body reaction category scores 0-4 (see Table 1) in the four treatments (CO = control group; LA = local anaesthetic; LT = intramuscular analgesic; LL = local anaesthetic plus intramuscular analgesic) on different sampling occasions (baseline, branding, and 5 days and 60 days after branding)

Treatment	Body reaction	Baseline score (%)					Score at branding (%)					Score at 5 days after branding (%)					Score at 60 days after branding (%)				
		0	1	2	3	4	0	1	2	3	4	0	1	2	3	4	0	1	2	3	4
**CO**	**Movement**	39.1	47.8	4.3	8.7	0.0	56.5	17.4	17.4	8.7	0.0	60.9	17.4	17.4	4.3	0.0	47.8	39.1	4.3	8.7	0.0
	**Tail position/movement**	4.3	34.8	47.8	8.7	4.3	0.0	8.7	21.7	52.2	17.4	13.0	65.2	17.4	0.0	4.3	0.0	52.2	34.8	13.0	0.0
	**Hind legs movement**	50.0	27.3	18.2	4.5	--	65.2	8.7	17.4	8.7	--	78.3	4.3	13.1	4.3	--	73.9	17.4	8.7	0.0	--
	**Movement of back/spine**	59.1	27.3	13.6	--	--	73.9	17.4	8.7	--	--	82.6	0.0	17.4	--	--	78.3	8.7	13.0	--	--
	**Reaction at release**	59.1	27.3	13.6	0,0	--	73,9	17.4	8.7	0.0	--	82.6	0.0	17.4	0.0	--	78.3	8.7	13.0	0.0	--
	**Body response to branding***	--	--	--	--	--	47.8	21.7	30.4	--	--	--	--	--	--	--	--	--	--	--	--
**LA**	**Movement**	65.2	26.1	4.3	4.3	0.0	56.5	34.8	8.7	0.0	0.0	65.2	21.7	13.0	0.0	0.0	47.8	30.4	17.4	4.3	0.0
	**Tail position/movement**	4.3	60.9	30.4	4.3	0.0	0.0	13.0	17.4	43.5	26.1	4.3	56.5	26.1	13.0	0.0	0.0	60.9	34.8	4.3	0.0
	**Hind legs movement**	57.1	33.3	4.8	4.8	--	69.6	13.0	17.4	0.0	--	87.0	13.0	0.0	0.0	--	78.3	8.7	13.0	0.0	--
	**Movement of back/spine**	86.7	4.8	9.5	--	--	65.2	8.7	26.1	--	--	90.9	4.5	4.6	--	--	78.3	13.0	8.7	--	--
	**Reaction at release**	85.7	4.8	9.5	0.0	--	65.2	8.7	26.1	0.0	--	87.0	8.7	4.3	0.0	--	78.3	13.0	8.7	0.0	--
	**Body response to branding***	--	--	--	--	--	39.1	43.5	17.4	--	--	--	--	--	--	--	--	--	--	--	--
**LL**	**Movement**	26.1	43.5	17.4	4.3	8.7	43.5	26.1	21.7	8.7	0.0	56.5	30.4	8.7	4.3	0.0	56.5	13.0	13.0	13.0	4.3
	**Tail position/movement**	0.0	30.4	60.9	4.3	4.3	0.0	17.4	17.4	52.2	13.0	4.3	56.5	39.1	0.0	0.0	0.0	52.2	43.5	4.3	0.0
	**Hind legs movement**	40.9	36.4	9.1	13.6	--	77.3	9.1	4.5	9.1	--	73.9	13.0	8.7	4.4	--	60.9	30.4	8.7	0.0	--
	**Movement of back/spine**	54.5	27.3	18.2	--	--	54.6	13.6	31.8	--	--	73.9	0.0	26.1	--	--	60.9	21.7	17.4	--	--
	**Reaction at release**	59.1	27.3	13.6	0.0	--	54.6	13.6	31.8	0.0	--	73.9	0.0	26.1	0.0	--	60.9	21.7	17.4	0.0	--
	**Body response to branding***	--	--	--	--	--	31.8	31.8	36.4	--	--	--	--	--	--	--	--	--	--	--	--
**LT**	**Movement**	39.1	43.5	4.3	13.0	0.0	52.2	17.4	21.7	8.7	0.0	56.5	17.4	21.7	4.3	0.0	43.5	34.8	17.4	4.3	0.0
	**Tail position/movement**	8.7	39.1	47.8	4.3	0.0	0.0	17.4	21.7	39.1	21.7	0.0	47.8	43.5	8.7	0.0	0.0	47.8	43.5	4.3	4.3
	**Hind legs movement**	57.2	23.8	9.5	9.5	--	60.9	17.4	13.0	8.7	--	69.6	13.0	17.4	0.0	--	71.4	19.1	9.5	0.0	--
	**Movement of back/spine**	61.9	9.5	28.6	--	--	43.5	21.7	34.8	--	--	78.3	13.0	8.7	--	--	76.2	14.3	9.5	--	--
	**Reaction at release**	61.9	9.5	28.6	0.0	--	43.5	21.7	34.8	0.0	--	78.3	13.0	8.7	0.0	--	76.2	14.3	9.5	0.0	--

**Body response to branding***	--	--	--	--	--	54.5	31.8	13.7	--	--	--	--	--	--	--	--	--	--	--	--

*Assessed only during branding.

Supplementary Table S2. Percentage distribution of facial expression category scores 0-2 (see Table 2) in the four treatments (CO = Control group; LA = local anaesthetic; LT = intramuscular analgesic; LL = local anaesthetic plus intramuscular analgesic) on different sampling occasions (baseline, branding, and 5 days and 60 days after branding)

		**Baseline score (%)**			**Score at branding (%)**			**Score at 5 days after branding (%)**			**Score at 60 days after branding (%)**		
**Treatment**	**Facial expression**	0	1	2	0	1	2	0	1	2	0	1	2
**CO**	**Head position**	13.1	21.7	65.2	30.5	21.7	47.8	26.1	26.1	47.8	17.4	34.8	47.8
	**Escape reaction**	13.1	65.2	21.7	30.4	52.2	17.4	26.1	60.9	13.0	17.4	69.6	13.0
	**Eye white showing**	8.7	91.3	--	21.7	78.3	--	21.7	78.3	--	0.0	100.0	--
	**Tension above eye**	0.0	56.5	43.5	8,7	60.9	30.4	4.4	39.1	56.5	13.0	65.2	21.8
	**Eye tightness**	43.5	17.4	39.1	26.1	34.8	39.1	34.8	34.8	30.4	82.6	8.7	8.7
	**Third eyelid**	39.1	60.9	--	47.8	52.2	--	43.5	56.5	--	56.5	43.5	--
	**Tension masticatory muscles**	0.0	56.5	43.5	8.7	60.9	30.4	4.3	39.1	56.5	13.0	65.2	21.7
	**Tension of the muzzle**	21.8	39.1	39.1	17.4	30.4	52.3	4.3	52.3	43.4	8.7	43.5	47.8
	**Opening mouth**	91.3	8.7	0.0	43.5	34.8	21.7	87.0	8.7	4.3	82.6	13.0	4.3
	**Face response to branding***	--	--	--	47,6	0,0	52,4	--	--	--	--	--	--
**LA**	**Head position**	22.7	18.2	59.1	31.8	22.7	45.5	17.4	30.4	52.2	21.7	13.1	65.2
	**Escape reaction**	31.8	54.6	13.6	31.8	54.6	13.6	30.4	60.9	8.7	21.7	69.6	8.7
	**Eye white showing**	9.1	90.9	--	18.2	81.8	--	4.4	95.6	--	0.0	100.0	--
	**Tension above eye**	4.6	54.5	40,9	13.6	59.1	27.3	4.4	65.2	30.4	21.7	60.9	17.4
	**Eye tightness**	45.4	36.4	18.2	50,0	27.3	22.7	60.9	26.1	13.0	69.6	21.7	8.7
	**Third eyelid**	54.5	45.5	--	77.3	22.7	--	60,9	39.1	--	60.9	39.1	--
	**Tension masticatory muscles**	13.0	52.2	34.8	56.5	21.7	21.7	39.1	52.2	8.7	30.4	52.2	17.4
	**Tension of the muzzle**	4.5	54.6	40,9	31.8	36.4	31.8	4.4	56.5	39.1	0.0	39.1	60.9
	**Opening mouth**	81.8	18.2	0.0	68.2	13.6	18.2	95.7	4.3	0.0	87.0	4.3	8.7
	**Face response to branding***	--	--	--	50,0	0,0	50,0	--	--	--	--	--	--

Supplementary Table S2 (contd.)

**LL**	**Head position**	4.6	22.7	73.7	26.1	21.7	52.2	17.4	21.7	60.9	8.7	13.0	78.3
	**Escape reaction**	4.5	68.2	27.3	34.8	47.8	17.4	26.1	47.8	26.1	21.7	52.2	26.1
	**Eye white showing**	9.1	90.0	--	17.4	82.6	--	17.4	82.6	--	8.7	91.3	--
	**Tension above eye**	4.5	40.9	54.6	4.3	60.9	34.8	8.7	47.8	43.5	8.7	78.3	13.0
	**Eye tightness**	54.6	13.6	31.8	34.8	39.1	26.1	47.8	21.7	30.5	69.6	30.4	0.0
	**Third eyelid**	68.2	31.8	--	56.5	43.5	--	73.9	26.1	--	65.2	34.8	--
	**Tension masticatory muscles**	26.1	39.1	34.8	39.1	21.7	39.1	43.5	17.4	39.1	30.4	34.8	34.8
	**Tension of the muzzle**	4.5	45.5	50,0	8.7	30.4	60.9	0.0	39.1	60.9	4.3	26.1	69.6
	**Opening mouth**	90.9	9.1	0.0	65.2	17.4	17.4	100.0	0.0	0.0	100.0	0.0	0.0
	**Face response to branding***	--	--	--	38,1	0,0	61,9	--	--	--	--	--	--
**LT**	**Head position**	4.6	31.8	63.6	21.7	21.8	56.5	21.7	8.7	69.6	4.7	14.3	81.0
	**Escape reaction**	9,1	63,6	27.3	26.1	43.5	30.4	21.7	52.2	26.1	9,5	57.2	33.3
	**Eye white showing**	0.0	100,0	--	4.3	95.7	--	8.7	91.3	--	4.5	95.5	--
	**Tension above eye**	4.5	50.0	45.5	8.7	56.5	34.8	4.4	47.8	47.8	4.8	57.1	38.1
	**Eye tightness**	40.9	31.8	27.3	40.9	27.3	31.8	52.2	13.0	34.8	71.4	23.8	4.8
	**Third eyelid**	54.5	45.5	--	60.9	39.1	--	56.5	43.5	--	52.4	47.6	--
	**Tension masticatory muscles**	13.0	56.5	30.4	47.8	26.1	26.1	30.4	30.4	39.1	39.1	43.5	17.4
	**Tension of the muzzle**	9.1	31.8	59.1	21.7	21.8	56.5	13.0	34.8	52.2	4.8	23.8	71.4
	**Opening mouth**	90,9	9.1	0.0	78.3	4.3	17.4	95.7	4.3	0.0	85.7	9.5	4.8
	**Face response to branding***	--	--	--	50.0	0.0	50.0	--	--	--	--	--	--

* Assessed only during branding.
